# 5-Methyl-2-nitro­aniline

**DOI:** 10.1107/S2414314625007473

**Published:** 2025-08-27

**Authors:** Lily Samson, Lydia Banwart, Sajan Silwal, Marcus R. Bond

**Affiliations:** a Department of Chemistry and Physics Southeast Missouri State University,Cape Girardeau MO 63701 USA; University of Aberdeen, United Kingdom

**Keywords:** crystal structure, hydrogen bonding, DFT geometry optimization

## Abstract

The bond lengths and angles within the title mol­ecule, C_7_H_8_N_2_O_2_, conform to average values for other 5-substituted-2-nitro­anilines, and with those calculated by a DFT geometry optimization. The short C—NH_2_ bond length of 1.3469 (12) Å is indicative of substantial involvement of the aniline N-atom in the aromatic π bonding system of the ring. In the extended structure, N—H⋯O hydrogen bonds link the mol­ecules into [001] tapes, which aggregate into zipper-like folded ribbons. Layers of parallel ribbons stack along *a* to complete the structure.

## Abstract 2

The short C—NH_2_ bond length of 1.3469 (12) Å in the title mol­ecule is indicative of substantial involvement of the aniline N-atom in the aromatic π bonding system of the ring. In the extended structure, N—H⋯O hydrogen bonds link the mol­ecules into [001] tapes, which aggregate into zipper-like folded ribbons. Layers of parallel ribbons stack along *a* to complete the structure.

## Graphical abstract

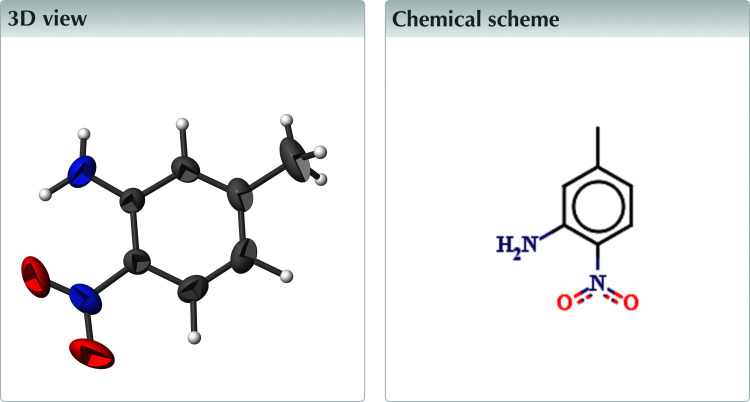

## Structure description

**Figure 1:**
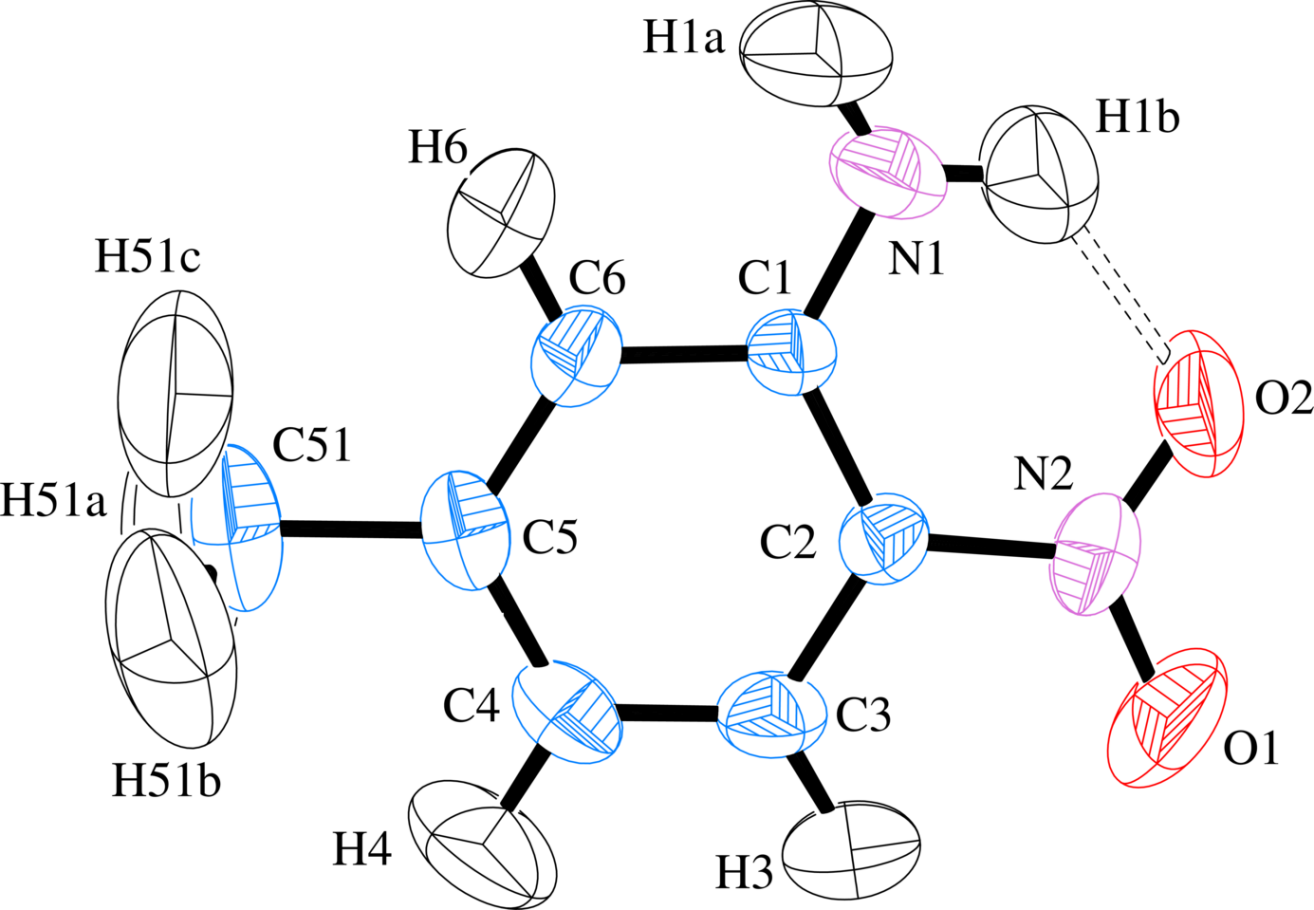
Displacement ellipsoid plot at the 50% probability level of (**I**) with atom labels. The intra­molecular N—H⋯O hydrogen bond is indicated by a dashed line.

The title mol­ecule, C_7_H_8_N_2_O_2_ (**I**), is approximately planar with the ring C atoms exhibiting an average deviation of 0.003 (2) Å from the mean plane (Fig. 1 ▸). The mol­ecular plane lies closest to the *bc* plane of the unit cell but is canted to form an angle of 17.25° between normals. The geometry about both N atoms is almost planar so that *sp*^2^ hybridization can be reasonably assigned. The aniline nitro­gen atom (N1) shows the most pyramidalization with a distance of 0.0792 (1) Å of this atom from the mean plane of its attached H atoms and C1, *versus* 0.0005 (1) Å for N2. The amino and nitro group planes make twist angles of 5.1 (7) and 3.87 (4)°, respectively, with respect to the mean plane of the C1–C6 ring. One H atom of the amine group makes an intra­molecular hydrogen bond to an O atom of the nitro group (Table 1 ▸). The nitro group geometry is uniform with the N—O distances agreeing within 1 s.u. of each other, and in agreement with the mean N—O bond length for 5-substituted-2-nitro­anilines reported in the Cambridge Structural Database [1.233 (23) Å; 35 hits, CSD version 5.45, June 2024 update; Groom *et al.*, 2016 ▸]. Other geometrical parameters agree well with average values from the CSD. The nitro group bond angles are close to 120° with the O—N—O angle slightly larger [121.20 (9)° for O—N—O and 119.4 (9)° for C—N—O for CSD mean values]. The C1—N1 bond length of 1.3469 (12) Å in (**I**) is significantly shorter than the sum of the covalent radii of 1.44 Å but similar to the CSD mean of 1.341 (25) Å for a C_ar_—NH_2_ bond, a familiar situation in aniline compounds where the nominal lone pair of the nitro­gen atom participates in the aromatic π-bonding network of the ring (Morrison & Boyd, 1976 ▸). The C2—N2 distance to the nitro group is only slightly shorter than the sum of the covalently radii [CSD mean = 1.422 (25) Å].

**Figure 2:**
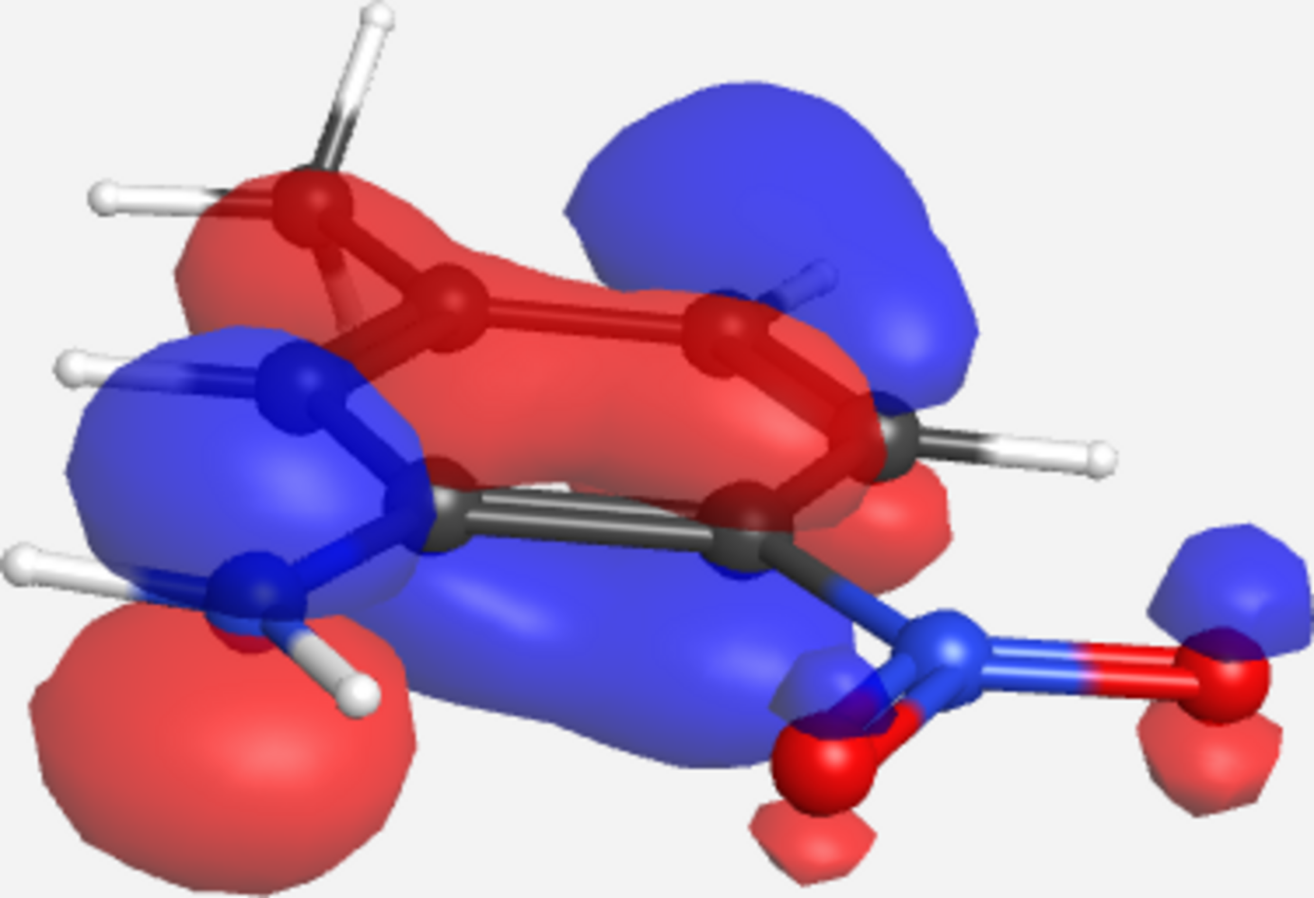
Plot of the highest occupied mol­ecular orbital for (**I**) from the DFT geometry optimization.

A DFT geometry optimization of the title mol­ecule *in vacuo* [B3LYP, 6311+G(d,p); *GAMESS* (Schmidt *et al.*, 1993 ▸)] provides geometric parameters in broad agreement with experimental values. Of note, the optimized aniline C—N distance is only 0.01 Å longer than the experimental value. In addition, there is significant contribution of the *p*-orbital on the N atom to the delocalized π-bonding system of the aromatic ring in the highest occupied mol­ecular orbital (HOMO), as shown in the plot in Fig. 2 ▸. The nitro group exhibits the greatest deviation from the experimental values with the C—N bond longer by 0.024 Å and unequal N—O bond lengths (the O atom involved in intra­molecular hydrogen bonding is 0.016 Å longer). A MOL file containing the optimized geometry is available in supporting information.

**Figure 3:**
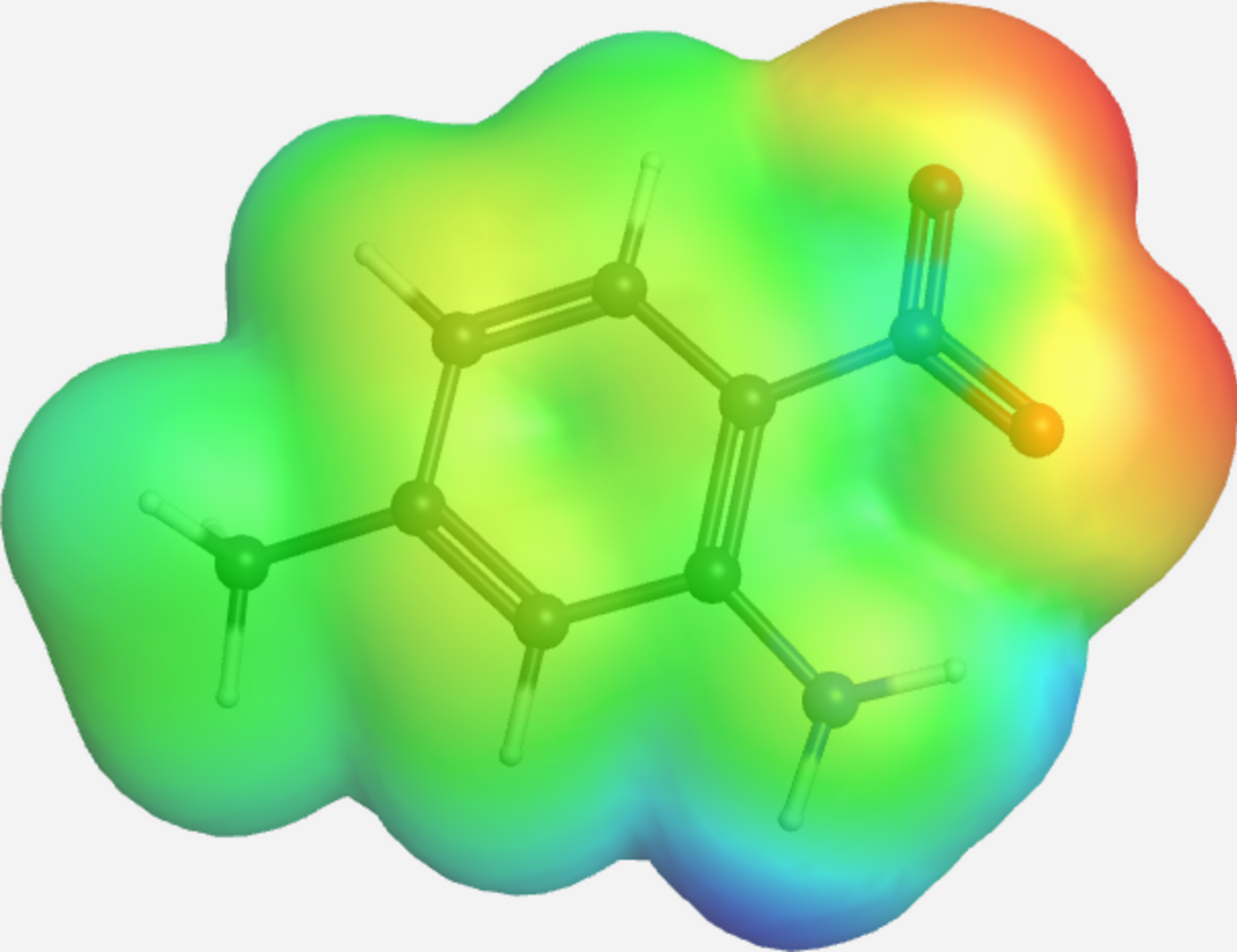
Electrostatic potential plot for (**I**) from the DFT geometry optimization. Red indicates accumulation of negative charge and blue accumulation of positive charge.

**Figure 4:**
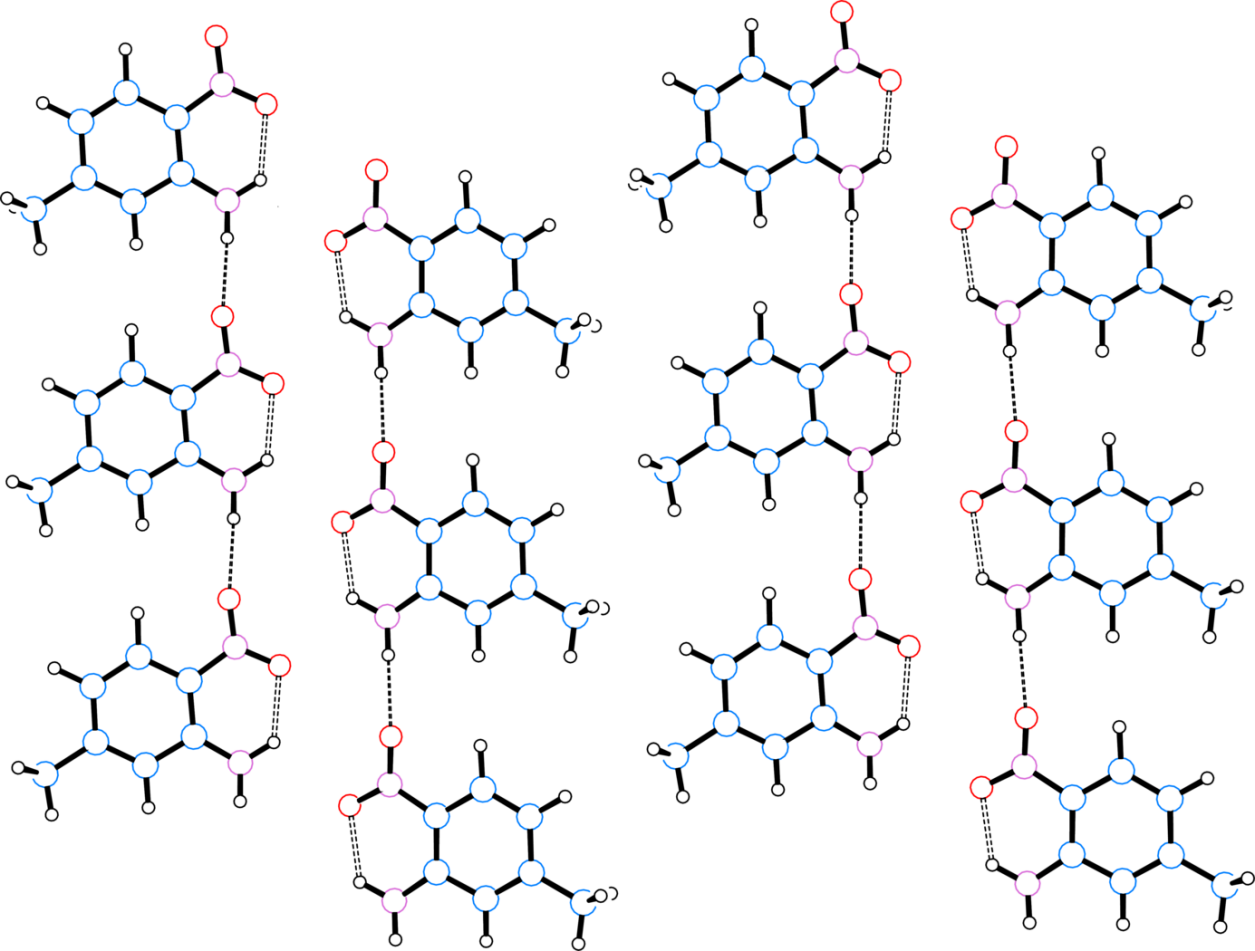
Packing diagram for a corrugated sheet of mol­ecules of (**I**) viewed down *a* with *b* horizontal and *c* vertical. Atoms are drawn as circles of arbitrary radii, intra­molecular hydrogen bonds are indicated by thick dashed lines, and inter­molecular hydrogen bonds are indicated by thin dashed lines.

**Figure 5:**
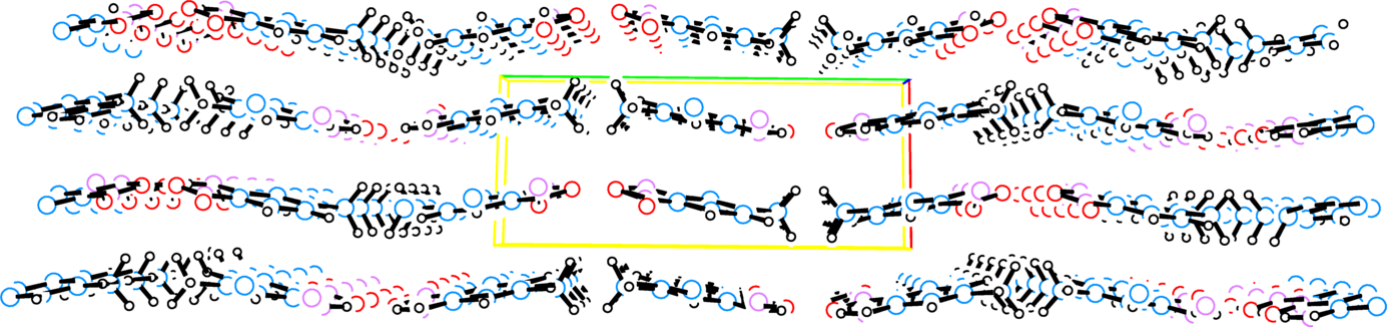
Unit-cell packing diagram for (**I**) viewed down *c* with *a* vertical and *b* horizontal. Atoms are drawn as circles of arbitrary radii.

In the extended structure of (**I**), inter­molecular hydrogen bonding between H1 and O1 of a neighboring mol­ecule links mol­ecules into tapes propagating parallel to *c*. The opposite polarity of the amino- and nitro- groups, as shown in the electrostatic potential plot in Fig. 3 ▸, generates a zipper-like folded ribbon between two neighboring tapes to place groups of opposite polarity in neighboring tapes in close proximity. The hydro­phobic methyl groups from neighboring ribbons abut to generate corrugated sheets in the *bc* plane. These sheets are stacked along *a* with only slight overlap between phenyl rings in neighboring sheets and with the fold of polar groups in one sheet overlying the fold of hydro­phobic groups in the neighboring sheets. A table of hydrogen-bond parameters is presented in Table 1 ▸, a packing diagram for a single sheet is presented in Fig. 4 ▸, and a unit-cell packing diagram is presented in Fig. 5 ▸.

Other known methyl-2-nitro­anilines exhibit polymorphs with different hydrogen-bonding arrangements. For the 4-methyl derivative, two polymorphs crystallized from different solvents are known. The first is in monoclinic *C*2/*c* with *Z′* = 1 (CSD refcode TEHGUI/02; Ellena *et al.*, 1996 ▸; Nigam & Murty, 1965 ▸; from ethanol) while the other is in triclinic *P*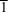 with *Z′* = 2 (TEHGUI01/03; Cannon *et al.*, 2001 ▸; Aguirre *et al.*, 2024 ▸; from acetone). The 6-methyl derivative likewise occurs in two polymorphs. Both crystallize in monoclinic *P*2_1_/*c* but differ in unit-cell volumes by a factor of approximately 2 [KEFYOK (Jing *et al.*, 2006 ▸) and KEFYOK01/KEFYOK02 (Callear & Hursthouse, 2009 ▸)]. The *Z*′ = 2 polymorph is crystallized from *N*,*N*-di­methyl­formamide (KEFYOK) or methanol/imidazolidine-2-thione (KEFYOK01) while the *Z*′ = 1 polymorph crystallizes from methanol/benzene­sulfonic acid. The structure of the 3-methyl derivative remains unreported while a systematic search for polymorphs of the title compound has not been conducted.

Bifurcated N—H⋯(O,O) hydrogen bonding from an amino proton to the nitro O atoms is a common motif in the structures of 2-nitro anilines. Asymmetric bifurcated hydrogen bonding is observed in KEFYOK/01 and TEHGUI, while a mix of symmetric, bifurcated hydrogen bonding and direct hydrogen bonding is found for symmetry-unique mol­ecules in TEHGUI01. Extended mol­ecular arrangements in these structures consist of spiral, square columns in KEFYOK/01 but planar layers in TEHGUI and TEHGUI01. The extended structure of KEFYOK02 provides the greatest similarity to the title compound with direct inter­molecular N—H⋯O hydrogen bonding linking mol­ecules into corrugated layers, albeit with hydrogen bonding now between mol­ecules on opposite sides of the corrugation fold. Placement of the methyl group adjacent to the amino group eliminates the need for a hydro­phobic fold, as in the title structure, but also results in a longer H⋯O hydrogen bond contact distance of 2.32 Å.

## Synthesis and crystallization

5-Methyl-2-nitro­aniline (99.9%, AmBeed) was recrystallized from ethanol solution by slow evaporation to yield diffraction-quality crystals.

## Refinement

Crystal data, data collection, and structure refinement details are listed in Table 2 ▸. Structure solution and initial refinement using an independent atom model occurred within the Bruker *SHELXTL* software package (Version 2016/6). A disordered model for the methyl H atoms resulted in a higher agreement factor, thus the ordered model was retained. Final structure refinement occurred within the *OLEX2*–1.5 system *via* Hirshfeld atom refinement using *NoSpherA2* (Kleemiss *et al.*, 2021 ▸; Midgley *et al.*, 2021 ▸) with non-spherical atomic form factors derived from electron density determined by DFT calculations using *ORCA 5.0* (B3LYP functional, def2-SVP basis set; Neese, 2022). All atoms were refined anisotropically. This resulted in a slightly underdetermined data:parameter ratio (9.83:1) as a consequence of pursuing the non-spherical refinement. Four low angle reflections with *F_o_ << F*_c_ were presumed to be blocked by the beam catcher and omitted from the refinement.

## Supplementary Material

Crystal structure: contains datablock(s) I. DOI: 10.1107/S2414314625007473/hb4532sup1.cif

Structure factors: contains datablock(s) I. DOI: 10.1107/S2414314625007473/hb4532Isup2.hkl

MOL file from quantum mechanical geometry optimization. DOI: 10.1107/S2414314625007473/hb4532sup3.mol

Supporting information file. DOI: 10.1107/S2414314625007473/hb4532Isup4.cml

CCDC reference: 2481714

Additional supporting information:  crystallographic information; 3D view; checkCIF report

## Figures and Tables

**Table 1:** Hydrogen-bond geometry (Å, °)

*D*—H⋯*A*	*D*—H	H⋯*A*	*D*⋯*A*	*D*—H⋯*A*
N1—H2⋯O2	0.987 (14)	1.934 (14)	2.6356 (15)	125.7 (10)
N1—H1⋯O1^i^	0.968 (13)	2.133 (13)	3.0897 (14)	169.4 (12)

Symmetry code: (i) 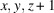.

**Table 2:** Experimental details

Crystal data	
Chemical formula	C_7_H_8_N_2_O_2_
*M*_r_	152.15
Crystal system, space group	Monoclinic, *P*2_1_/*c*
Temperature (K)	295
*a*, *b*, *c* (Å)	7.4151 (7), 15.8116 (16), 7.1225 (7)
β (°)	118.524 (3)
*V* (Å^3^)	733.71 (13)
*Z*	4
Radiation type	Mo *K*α
μ (mm^−1^)	0.10
Crystal size (mm)	0.23 × 0.21 × 0.10

Data collection	
Diffractometer	Bruker APEXII CCD
Absorption correction	Multi-scan (*SADABS*; Krause *et al.*, 2015 ▸)
*T*_min_, *T*_max_	0.670, 0.746
No. of measured, independent and observed [*I* ≥ 2σ(*I*)] reflections	19599, 1700, 1311
*R*_int_	0.035
(sin θ/λ)_max_ (Å^−1^)	0.651

Refinement	
*R*[*F*^2^ > 2σ(*F*^2^)], *wR*(*F*^2^), *S*	0.033, 0.055, 1.12
No. of reflections	1700
No. of parameters	173
H-atom treatment	All H-atom parameters refined
Δρ_max_, Δρ_min_ (e Å^−3^)	0.18, −0.13

Computer programs: *APEX3* and *SAINT* (Bruker, 2017 ▸), *SHELXT2018/2* (Sheldrick, 2015*a* ▸), *OLEX2.refine* (Bourhis *et al.*, 2015 ▸) *SHELXL2018/3* (Sheldrick, 2015*b* ▸), *ORTEP-3 for Windows* 2020.1 (Farrugia, 2012 ▸), *ORTEPIII* (Burnett & Johnson, 1996 ▸), *OLEX2* (Dolomanov *et al.*, 2009 ▸), *publCIF* (Westrip, 2010 ▸) and *PARST* (Nardelli, 1995 ▸).

## full crystallographic data

### 5-Methyl-2-nitroaniline Crystal data

**Table:**

C_7_H_8_N_2_O_2_	*F*(000) = 320.241
*M**_r_* = 152.15	*D*_x_ = 1.377 Mg m^−^^3^
Monoclinic, *P*2_1_/*c*	Mo *K*α radiation, λ = 0.71073 Å
*a* = 7.4151 (7) Å	Cell parameters from 7279 reflections
*b* = 15.8116 (16) Å	θ = 2.6–26.9°
*c* = 7.1225 (7) Å	µ = 0.10 mm^−^^1^
β = 118.524 (3)°	*T* = 295 K
*V* = 733.71 (13) Å^3^	Irregular, yellow
*Z* = 4	0.23 × 0.21 × 0.10 mm

### 5-Methyl-2-nitroaniline Data collection

**Table:**

Bruker APEXII CCD diffractometer	1311 reflections with *I*≥ 2σ(*I*)
φ and ω scans	*R*_int_ = 0.035
Absorption correction: multi-scan (SADABS; Krause *et al.*, 2015)	θ_max_ = 27.6°, θ_min_ = 3.5°
*T*_min_ = 0.670, *T*_max_ = 0.746	*h* = −9→9
19599 measured reflections	*k* = −20→20
1700 independent reflections	*l* = −9→9

### 5-Methyl-2-nitroaniline Refinement

**Table:**

Refinement on *F*^2^	Primary atom site location: dual
Least-squares matrix: full	All H-atom parameters refined
*R*[*F*^2^ > 2σ(*F*^2^)] = 0.033	*w* = 1/[σ^2^(*F*_o_^2^) + (0.0097*P*)^2^ + 0.0677*P*] where *P* = (*F*_o_^2^ + 2*F*_c_^2^)/3
*wR*(*F*^2^) = 0.055	(Δ/σ)_max_ = 0.0004
*S* = 1.12	Δρ_max_ = 0.18 e Å^−^^3^
1700 reflections	Δρ_min_ = −0.13 e Å^−^^3^
173 parameters	Extinction correction: Zachariasen, Fc^*^=kFc[1+0.001xFc^2^λ^3^/sin(2θ)]^-1/4^
0 restraints	Extinction coefficient: 0.027 (3)
0 constraints	

### 5-Methyl-2-nitroaniline Fractional atomic coordinates and isotropic or equivalent isotropic displacement parameters (Å^2^)

**Table:**

	*x*	*y*	*z*	*U*_iso_*/*U*_eq_	
N1	0.66254 (18)	0.62975 (7)	0.90340 (19)	0.0615 (3)	
H1	0.675 (2)	0.6288 (8)	1.045 (2)	0.091 (5)	
H2	0.661 (2)	0.6839 (9)	0.834 (2)	0.084 (5)	
C1	0.70816 (13)	0.55938 (5)	0.82802 (12)	0.0392 (2)	
C2	0.73877 (13)	0.55572 (5)	0.64725 (12)	0.0396 (2)	
N2	0.72344 (13)	0.62938 (6)	0.52484 (12)	0.0551 (2)	
O1	0.76280 (14)	0.62386 (6)	0.37703 (13)	0.0905 (3)	
O2	0.67125 (14)	0.69659 (5)	0.56884 (13)	0.0803 (3)	
C3	0.78714 (15)	0.47926 (6)	0.58181 (16)	0.0496 (3)	
H3	0.8103 (18)	0.4805 (7)	0.4418 (18)	0.082 (4)	
C4	0.80415 (16)	0.40613 (7)	0.69101 (17)	0.0553 (3)	
H4	0.842 (2)	0.3482 (7)	0.6426 (19)	0.100 (4)	
C5	0.77246 (14)	0.40677 (6)	0.87032 (15)	0.0503 (3)	
C51	0.7879 (3)	0.32578 (11)	0.9867 (3)	0.0807 (5)	
H51a	0.792 (4)	0.3353 (11)	1.130 (3)	0.152 (9)	
H51b	0.669 (3)	0.2851 (10)	0.901 (3)	0.168 (9)	
H51c	0.927 (3)	0.2944 (10)	1.030 (4)	0.149 (8)	
C6	0.72643 (15)	0.48186 (6)	0.93469 (15)	0.0459 (2)	
H6	0.7037 (17)	0.4837 (6)	1.0718 (17)	0.077 (3)	

### 5-Methyl-2-nitroaniline Atomic displacement parameters (Å^2^)

**Table:**

	*U*^11^	*U*^22^	*U*^33^	*U*^12^	*U*^13^	*U*^23^
N1	0.0848 (7)	0.0497 (6)	0.0571 (6)	0.0121 (5)	0.0395 (6)	−0.0076 (5)
H1	0.125 (12)	0.087 (10)	0.073 (9)	0.007 (8)	0.058 (9)	−0.021 (8)
H2	0.106 (11)	0.064 (9)	0.078 (10)	0.014 (9)	0.042 (8)	0.012 (8)
C1	0.0478 (5)	0.0363 (5)	0.0351 (4)	0.0018 (4)	0.0212 (4)	−0.0017 (4)
C2	0.0451 (5)	0.0400 (5)	0.0341 (4)	−0.0007 (4)	0.0193 (4)	0.0004 (4)
N2	0.0615 (6)	0.0553 (5)	0.0456 (5)	−0.0038 (4)	0.0231 (4)	0.0142 (4)
O1	0.1153 (7)	0.1071 (7)	0.0678 (5)	0.0005 (5)	0.0588 (5)	0.0299 (5)
O2	0.1099 (7)	0.0440 (4)	0.0839 (6)	0.0059 (4)	0.0437 (5)	0.0219 (4)
C3	0.0590 (6)	0.0520 (6)	0.0431 (5)	0.0012 (5)	0.0286 (5)	−0.0096 (5)
H3	0.110 (10)	0.086 (9)	0.074 (8)	0.006 (7)	0.064 (8)	−0.011 (7)
C4	0.0630 (7)	0.0389 (6)	0.0619 (6)	0.0029 (5)	0.0282 (5)	−0.0108 (5)
H4	0.131 (11)	0.059 (8)	0.113 (10)	0.013 (7)	0.060 (9)	−0.027 (7)
C5	0.0496 (5)	0.0367 (5)	0.0575 (6)	−0.0019 (4)	0.0199 (5)	0.0058 (4)
C51	0.0763 (11)	0.0490 (9)	0.1010 (12)	−0.0029 (7)	0.0296 (10)	0.0278 (8)
H51a	0.23 (2)	0.089 (13)	0.143 (16)	−0.003 (14)	0.093 (17)	0.043 (12)
H51b	0.113 (14)	0.101 (12)	0.20 (2)	−0.055 (11)	0.005 (12)	0.060 (12)
H51c	0.102 (14)	0.101 (12)	0.24 (2)	0.036 (10)	0.075 (14)	0.080 (13)
C6	0.0538 (6)	0.0460 (6)	0.0412 (5)	0.0000 (4)	0.0252 (5)	0.0061 (4)
H6	0.106 (9)	0.073 (8)	0.066 (7)	0.005 (6)	0.051 (7)	0.019 (6)

### 5-Methyl-2-nitroaniline Geometric parameters (Å, º)

**Table:**

N1—H1	0.968 (13)	C3—C4	1.3653 (14)
N1—H2	0.987 (14)	C4—H4	1.062 (10)
N1—C1	1.3469 (12)	C4—C5	1.4046 (14)
C1—C2	1.4113 (11)	C5—C51	1.4997 (16)
C1—C6	1.4141 (12)	C51—H51a	1.021 (19)
C2—N2	1.4265 (11)	C51—H51b	1.025 (15)
N2—O1	1.2225 (10)	C51—H51c	1.048 (16)
N2—O2	1.2220 (11)	C5—C6	1.3732 (13)
C2—C3	1.4025 (12)	C6—H6	1.068 (10)
C3—H3	1.090 (10)		
			
H1—N1—H2	120.7 (11)	C3—C4—H4	120.8 (7)
C1—N1—H1	119.0 (8)	C5—C4—H4	119.1 (7)
C1—N1—H2	117.5 (8)	C3—C4—C5	120.12 (10)
C2—C1—N1	125.31 (9)	C4—C5—C51	119.64 (13)
C6—C1—N1	118.67 (9)	C6—C5—C51	121.29 (13)
C6—C1—C2	116.02 (8)	C4—C5—C6	119.08 (9)
C1—C2—N2	121.56 (8)	C5—C51—H51a	112.7 (11)
C1—C2—C3	121.11 (8)	C5—C51—H51b	113.0 (9)
C3—C2—N2	117.32 (8)	C5—C51—H51c	111.8 (9)
O1—N2—C2	119.07 (9)	H51a—C51—H51b	107.0 (14)
O2—N2—C2	119.73 (8)	H51a—C51—H51c	102.8 (14)
O1—N2—O2	121.20 (9)	H51b—C51—H51c	108.9 (14)
C2—C3—H3	117.7 (6)	C5—C6—C1	123.05 (9)
C4—C3—H3	121.6 (6)	C1—C6—H6	116.9 (5)
C2—C3—C4	120.62 (9)	C5—C6—H6	120.1 (5)
			
N1—C1—C2—N2	0.08 (11)	C1—C6—C5—C4	0.30 (11)
N1—C1—C2—C3	179.60 (10)	C1—C6—C5—C51	−179.25 (12)
N1—C1—C6—C5	−179.98 (9)	C2—C3—C4—C5	0.23 (11)
C1—C2—N2—O1	175.85 (9)	C3—C4—C5—C51	178.91 (12)
C1—C2—N2—O2	−4.24 (10)	C3—C4—C5—C6	−0.65 (12)
C1—C2—C3—C4	0.54 (10)		

### 5-Methyl-2-nitroaniline Hydrogen-bond geometry (Å, º)

**Table:**

*D*—H···*A*	*D*—H	H···*A*	*D*···*A*	*D*—H···*A*
N1—H2···O2	0.987 (14)	1.934 (14)	2.6356 (15)	125.7 (10)
N1—H1···O1^i^	0.968 (13)	2.133 (13)	3.0897 (14)	169.4 (12)

Symmetry code: (i) *x*, *y*, *z*+1.
